# Detecting acute neurotoxicity during platinum chemotherapy by neurophysiological assessment of motor nerve hyperexcitability

**DOI:** 10.1186/1471-2407-10-451

**Published:** 2010-08-23

**Authors:** Andrew Hill, Peter Bergin, Fritha Hanning, Paul Thompson, Michael Findlay, Dragan Damianovich, Mark J McKeage

**Affiliations:** 1 Cancer Clinical Pharmacology Research Group, School of Medical Sciences, Faculty of Medical and Health Sciences, University of Auckland, Auckland, New Zealand; 2 Departments of Neurophysiology, Auckland City Hospital, Auckland, New Zealand; 3 Department of Medical Oncology, Auckland City Hospital, Auckland, New Zealand

## Abstract

**Background:**

Platinum-based drugs, such as cisplatin and oxaliplatin, are well-known for inducing chronic sensory neuropathies but their acute and motor neurotoxicities are less well characterised. Use was made of nerve conduction studies and needle electromyography (EMG) to assess motor nerve excitability in cancer patients during their first treatment cycle with platinum-based chemotherapy in this study.

**Methods:**

Twenty-nine adult cancer patients had a neurophysiological assessment either before oxaliplatin plus capecitabine, on days 2 to 4 or 14 to 20 after oxaliplatin plus capecitabine, or on days 2 to 4 after carboplatin plus paclitaxel or cisplatin, undertaken by a neurophysiologist who was blinded to patient and treatment details. Patients completed a symptom questionnaire at the end of the treatment cycle.

**Results:**

Abnormal spontaneous high frequency motor fibre action potentials were detected in 100% of patients (n = 6) and 72% of muscles (n = 22) on days 2 to 4 post-oxaliplatin, and in 25% of patients (n = 8) and 13% of muscles (n = 32) on days 14 to 20 post-oxaliplatin, but in none of the patients (n = 14) or muscles (n = 56) tested prior to oxaliplatin or on days 2 to 4 after carboplatin plus paclitaxel or cisplatin. Repetitive compound motor action potentials were less sensitive and less specific than spontaneous high frequency motor fibre action potentials for detection of acute oxaliplatin-induced motor nerve hyperexcitability but were present in 71% of patients (n = 7) and 32% of muscles (n = 32) on days 2 to 4 after oxaliplatin treatment. Acute neurotoxicity symptoms, most commonly cold-induced paraesthesiae and jaw or throat tightness, were reported by all patients treated with oxaliplatin (n = 22) and none of those treated with carboplatin plus paclitaxel or cisplatin (n = 6).

**Conclusions:**

Abnormal spontaneous high frequency motor fibre activity is a sensitive and specific endpoint of acute oxaliplatin-induced motor nerve hyperexcitability, detectable on EMG on days 2 to 4 post-treatment. Objective EMG assessment of motor nerve excitability could compliment patient-reported symptomatic endpoints of acute oxaliplatin-induced neurotoxicity in future studies.

## Background

Cancer chemotherapy based on cisplatin, carboplatin and oxaliplatin, usually combined with radiation, capecitabine, paclitaxel or other agents, is the mainstay of treatment for many cancer patients, including those with lung, colorectum, stomach, head and neck, cervix, oesophagus, bladder, ovary and testis cancer [1]. However, platinum-induced chronic neuro-sensory toxicities are treatment-limiting, in particular glove and stocking distal paraesthesiae and dysesthesiae with loss of tendon reflexes and peripheral sensation, and ototoxicity with tinnitus and hearing loss [2-4]. Neuro-sensory toxicities typically develop after repeated dosing, progress cumulatively with continued treatment and persist long beyond the completion of treatment. In addition, oxaliplatin has been reported to cause a distinct acute and reversible neurotoxicity syndrome with motor symptoms [3], which has not been reported with other platinum-based drugs to date. While the chronic neuro-sensory toxicities of platinum-based drugs are well known, their acute and motor neurotoxicity are less well characterised.

In a previous study of cancer patients treated with oxaliplatin [5,6], striking neurophysiological abnormalities were demonstrated suggesting a state of acute peripheral nerve hyperexcitability due to altered axonal ion channel activity. These neurophysiological abnormalities included repetitive compound motor action potentials (CMAPs) during nerve conduction studies after a single electrical stimulus following the main CMAP, and high frequency motor fibre action potentials on needle electromyography (EMG) that occurred spontaneously and upon needle insertion or voluntary muscle contraction. Since these original reports, few or no subsequent studies by other groups have independently confirmed the occurrence of repetitive CMAPs and/or abnormal motor fibre action potentials in patients treated with oxaliplatin or other chemotherapy drugs.

In the current study, use was made of nerve conduction studies and EMG to assess motor nerve excitability in cancer patients during their first treatment cycle of platinum-based chemotherapy. We sought to define clinical procedures for the objective assessment of acute oxaliplatin-induced motor nerve hyperexcitability by optimising the time-points and endpoints for its neurophysiological detection. In addition, we aimed to independently confirm whether repetitive CMAPs and/or abnormal motor fibre action potentials were detectable after oxaliplatin treatment, and to obtain preliminary evidence of their occurrence after treatment with other platinum drugs. Neurophysiological studies are inherently subjective as they require qualitative interpretation. To reduce observer bias in the current study, a control group of patients was included who were tested prior to receiving platinum-based chemotherapy and the neurophysiologist was blinded to patient group and treatment details.

## Methods

### Patients

Eligible subjects were adult cancer patients who were to receive standard chemotherapy with oxaliplatin (130 mg/m^2^), cisplatin (100 mg/m^2^) or carboplatin (AUC 6 mg*min/ml) alone or in combination with other agents repeated three weekly, and who had given written informed consent. Subjects who had previously received platinum-based chemotherapy and those with previous or existing peripheral neuropathy or medical contraindications to clinical neurophysiological procedures were ineligible. The study was approved by the Auckland Ethics Committee × (Approval no AKX 04/06/165).

### Study Procedures and Groups

Study procedures included neurophysiological assessment and symptom evaluation both carried out during the first treatment cycle of platinum-based chemotherapy. The neurophysiological assessment was carried out once only to avoid subject drop-out, which was associated with repeated assessments in previous studies [6]. Study patients were allocated to one of four groups according to their treatment and the timing of their neurophysiological assessment, as follows: group 1, assessment prior to oxaliplatin; group 2, assessment on days 2 to 4 after oxaliplatin; group 3, assessment on days 14 to 20 after oxaliplatin, and; group 4, assessment on days 2 to 4 after carboplatin plus paclitaxel or cisplatin.

### Neurophysiological assessment

The neurophysiological assessment included a standard neurological examination, nerve conduction studies and EMG, carried out by a neurology specialist (PB) who was blinded to the patient and treatment details. To maintain blinding, the neurologist did not take a neurological history and ensured that patients did not volunteer any neurological symptoms.

The neurophysiological assessment was similar to that previously reported [5,6]. Standard nerve conduction techniques, electrode positions and stimulation sites were used. Limb temperature was monitored and maintained above 32°C. Neurophysiological testing was carried out in the limbs opposite to the arm used for the chemotherapy infusion. Sensory studies comprised sural, radial, median and ulnar sensory nerve action potentials (SNAPs). Motor studies were performed in median, ulnar, peroneal and tibial nerves. The presence of repetitive compound muscle action potentials (CMAPs) was assessed in the four motor nerves tested using an F-wave programme, with a slower sweep speed than conventional CMAP recordings. EMG was performed in first dorsal interosseous (FDIO), extensor digitorum communis (EDC), tibialis anterior and gastrocnemius muscles. Most patients had all 4 sensory and motor nerve studies performed, and all 4 muscles sampled by EMG, though a few did not have the complete set of these tests.

A simple scoring system for increased motor unit activity was created:

0 No abnormal motor unit activity

1 Increased insertional activity

2 Spontaneous high frequency motor unit activity with muscle clinically at rest, with bursts lasting for duration of less than 2 seconds

3 Spontaneous high frequency motor unit activity with muscle clinically at rest, with bursts lasting for duration of 2 to 5 seconds

4 Spontaneous high frequency motor unit activity with muscle clinically at rest, with bursts lasting for duration of more than 5 seconds

### Symptom evaluation

Patients completed a short symptom questionnaire, immediately before their neurophysiological assessment and at the end of the first treatment cycle, documenting the presence, absence, severity, onset time and duration of jaw or throat tightness, perioral paraesthesiae, muscle cramps, cold-induced paraesthesiae, glove and stocking numbness and tingling, infusional arm discomfort, loss of sensation of breathing, visual disturbance and any other neurotoxicity symptoms. The neurologist (PB) remained blinded to the result of this questionnaire. Symptom data from one oxaliplatin patient was unavailable because of sudden cardiac death.

### Data handling

A group size of 6 to 8 subjects was considered adequate to provide sufficient data for each time point and treatment group to give a reasonable estimate of the proportion of patients with motor nerve hyperexcitability. Data were analysed using descriptive statistics and reported as the number and proportion of patients, muscles and nerves affected by motor nerve hyperexcitability for each group. The standard error of each proportion was calculated as follows; standard error = √p(1-p)/n), where p is proportion and n is sample size. No formal evaluation of the statistical significance of differences between groups was planned or carried out. Test sensitivity was calculated from the number of patients, muscles or nerves displaying motor nerve hyperexcitability expressed as a percentage of the total number of patients, muscles or nerves tested on days 2 to 4 after oxaliplatin. Test specificity was calculated as the number of patients, muscles or nerves not displaying motor nerve hyperexcitability expressed as a percentage of the total number of patients, muscles or nerves tested before oxaliplatin or on days 2 to 4 after carboplatin plus paclitaxel or cisplatin.

## Results

### Patients and Treatment

The study population (Table 1) included a total of 29 patients (22 males and 7 females) ranging in age from 28 to 79 years (median 62 years), and cancer diagnoses included colorectal cancer Dukes C (13 patients), colorectal cancer metastatic (10 patients), head and neck cancer (4 patients) and non-small cell lung cancer (2 patients). All patients had ECOG performance status of 0 or 1. Eleven patients had one or more co-morbidities, most commonly hypertension (6 patients) or hypercholesterolaemia (3 patients). Seven patients had received prior chemotherapy, one regimen in 5 patients and two regimens in 2 patients, including 5-fluorouracil plus folinic acid (4 patients), single agent capecitabine (3 patients) and capecitabine plus irinotecan (2 patients). None of the patients had been previously treated with platinum-based chemotherapy. The study was undertaken during the first treatment cycle of platinum-based chemotherapy with oxaliplatin (130 mg/m^2 ^day 1) plus capecitabine (1000 to 2000 mg/m^2 ^bd po days 1 to 14) [23 patients], cisplatin (100 mg/m^2^) [4 patients] or carboplatin (AUC 6 mg*min/ml) plus paclitaxel (200 mg/m^2^) [2 patients].

**Figure 1:**
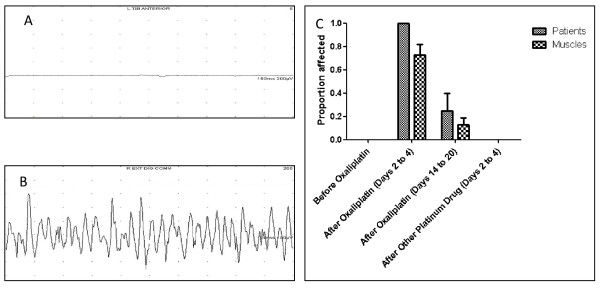
**Spontaneous high frequency motor fibre action potentials**. (A) Normal EMG at baseline; (B) EMG showing spontaneous high frequency motor fibre action potentials 46 hrs after oxaliplatin; (C) Proportion of patients and muscles affected by spontaneous high frequency motor fibre action potentials by time relative to chemotherapy.

**Table 1:** Patient Characteristics

		Number of Patients
**Age**	Median	62 years
	
	Range	28 to 79 years

**Gender**	Male	22
	
	Female	7

**Cancer Diagnosis**	Colorectal Dukes C	13
	
	Colorectal Metastatic	10
	
	Head and Neck	4
	
	Non-Small Cell Lung Cancer	2

**Co-morbidities**	None	18
	
	One or more^1^	11

**Prior chemotherapy**	None	22
	
	One or more regimens^2^	7

**Study Chemotherapy**	Oxaliplatin (130 mg/m^2^) with Capecitabine (1000 to 2000 mg BID po days 1 to 14)	23
	
	Cisplatin (100 mg/m^2^)	4
	
	Carboplatin (AUC 6 mg*min/ml) with Paclitaxel (200 mg/m^2^)	2

^1^hypertension (6 patients), hypercholesterolaemia (3 patients), goitre, rheumatoid arthritis, gout, benign prostatic hypertrophy, impaired glucose tolerance, ischaemic heart disease, polycythaemia rubra vera; renal stones, duodenal ulcer (1 patient each). ^2 ^5-fluorouracil plus folinic acid (4 patients), single agent capecitabine (3 patients), capecitabine plus irinotecan (2 patients).

### Spontaneous high frequency motor unit activity

EMGs were graded according to a study-specific scale shown in the methods section. Normal or increased insertional motor unit activity (grades 0 or 1) was the maximum severity grade of motor nerve hyperexcitability in all subjects having EMG testing prior to oxaliplatin or after carboplatin plus paclitaxel or cisplatin. An example of a normal EMG from a patient tested before any treatment is shown in Fig 1A. Abnormal, spontaneous high frequency motor unit activity (grade 2 or more) was found in 8 of 14 (57%) patients after oxaliplatin treatment but not in any other patient group (Table 2; Figure 1C). An example of an abnormal EMG showing spontaneous high frequency motor unit activity in a patient treated 46 hours earlier with oxaliplatin is shown in Figure 1B.

**Table 2:** Comparison of neurophysiological endpoints of acute oxaliplatin-induced motor nerve hyperexcitability

Number of Abnormal Tests/Total Number of Tests (%)		Spontaneous High Frequency Motor Unit Action Potentials	Repetitive Compound Motor Action Potentials
**Before**	Patients	0/8 (0%)	1/8 (13%)
	
**Oxaliplatin**	Muscles or Nerves	0/32 (0%)	1/32 (3.2%)

**After Oxaliplatin**	Patients	6/6 (100%)	5/7 (71%)
	
**(Days 2-4)**	Muscles or Nerves	16/22 (72%)	9/28 (32%)

**After Oxaliplatin**	Patients	2/8 (25%)	2/8 (25%)
	
**(Days 14-20)**	Muscles or Nerves	4/32 (12%)	3/31 (10%)

**After Other**	Patients	0/6 (0%)	1/6 (17%)
	
**Platinum Drug** **(Days 2-4)**	Muscles or Nerves	0/24 (0%)	2/24 (8%)

Sensitivity*	Patients	100%	71%
	
	Muscles or Nerves	72%	32%

Specificity*	Patients	100%	86%
	
	Muscles or Nerves	100%	95%

*For detecting oxaliplatin-induced motor nerve hyperexcitability on days 2 to 4 post-treatment

Abnormal, spontaneous high frequency motor unit activity was detected in all patients tested on days 2 to 4 after oxaliplatin (6 of 6; 100%) but in fewer patients tested on days 14 to 20 after oxaliplatin (2 of 8; 25%) and no patients tested prior to oxaliplatin (0 of 8; 0%), or on days 2 to 4 after carboplatin plus paclitaxel or cisplatin (0 of 6; 0%). Abnormal, spontaneous high frequency motor unit activity was detected in most muscles tested on days 2 to 4 after oxaliplatin (16/22; 72%) but in fewer muscles tested on days 14 to 20 after oxaliplatin (4 of 32; 12%) and in none of the muscles tested prior to oxaliplatin (0 of 32; 0%), or on days 2 to 4 after carboplatin plus paclitaxel or cisplatin (0 of 24; 0%).

The sensitivity of spontaneous high frequency motor unit activity for detecting acute motor nerve hyperexcitability on days 2 to 4 after oxaliplatin was 100% and 72% for patients and muscles respectively, and its specificity was 100% for both patients and muscles (Table 2).

No abnormalities in sensory nerve conduction studies were seen.

### Repetitive CMAPs

**Figure 2:**
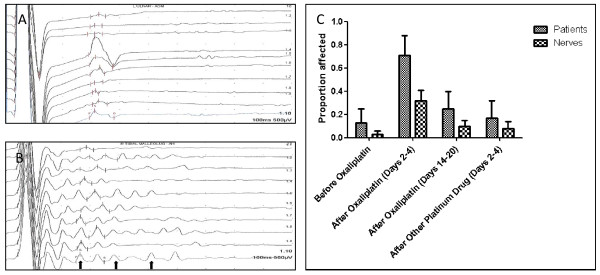
**Repetitive compound motor action potentials (CMAPs)**. (A) Normal CMAPs at baseline; (B) Repetitive CMAPs 46 hrs after oxaliplatin; (C) Proportion of patients and nerves affected by repetitive CMAPs by time relative to chemotherapy.

The presence of repetitive CMAPs was recorded as an all-or-nothing phenomenon in the absence of a method of grading the severity or extent of this abnormality. Abnormal repetitive CMAPs were found more frequently after oxaliplatin treatment but also occurred in groups not receiving oxaliplatin (Table 2; Figure 2). An example of a normal CMAP tracing from a patient tested prior to any treatment is shown in Figure 2A. A representative study showing abnormal repetitive CMAPs from a patient tested 46 hours after treatment with oxaliplatin is shown in Fig2B.

Repetitive CMAPs (Fig 2C) were present in most patients tested on days 2 to 4 after oxaliplatin (5 of 7; 71%) but in fewer patients tested before oxaliplatin (1 of 8; 13%), on days 14 to 20 after oxaliplatin (2 of 8; 25%) or on days 2 to 4 after carboplatin plus paclitaxel or cisplatin (1 of 6; 17%). Repetitive CMAPs were present in about one third of the nerves tested on days 2 to 4 after oxaliplatin (9 of 28; 32%) but in fewer nerves tested before oxaliplatin (1 of 32; 3.2%), on days 14 to 20 after oxaliplatin (3 of 31; 10%) or on days 2 to 4 after carboplatin plus paclitaxel or cisplatin (2 of 24; 8%).

The sensitivity of repetitive CMAPs for detecting acute motor nerve hyperexcitability on days 2 to 4 after oxaliplatin was 71% and 32% for patients and nerves, respectively. The specificity of repetitive CMAPs for detecting acute motor nerve hyperexcitability on days 2 to 4 after oxaliplatin was 86% and 95% for patients and nerves, respectively (Table 2).

### Neurotoxicity symptoms

Cold-induced paraesthesiae were reported by all patients treated with oxaliplatin (22 of 22; 100%) with its onset ranging from 0 to 48 hours post-treatment (median 0 hours) and duration ranging from 3 to 21 days (median 8 days). Jaw or throat tightness was reported by 15 of 22 patients (68%) treated with oxaliplatin with its onset ranging from 0 to 26 hours post-oxaliplatin (median 2 hours) and duration ranging from < 1 to 21 days (median 6 days). Pain in the upper limb used for the oxaliplatin infusion was reported by 12 of 22 patients (55%) treated with oxaliplatin but only in those receiving the drug via peripheral vein canula and in no patients given oxaliplatin via central venous line. Infusion arm discomfort came on 0 to 2 hours after oxaliplatin infusion (median 0 hours) and lasted from less than 1 day to 19 days (median 7.5 days). Other symptoms reported by oxaliplatin-treated patients included glove and stocking paraesthesiae unrelated to cold exposure (8 patients), muscle cramps (4 patients), transient change in vision (2 patients) and shortness of breath (1 patient).

None of the patients treated with carboplatin plus paclitaxel or cisplatin reported cold-induced paraesthesiae, jaw or throat tightness, infusion arm discomfort, glove and stocking paraesthesiae unrelated to cold exposure, muscle cramps, transient change in vision or shortness of breath.

## Discussion

In this study, we showed that abnormal, spontaneous high frequency motor unit action potentials were a sensitive and specific endpoint of acute oxaliplatin-induced motor nerve hyperexcitability. These abnormal, spontaneous high frequency motor unit action potentials, which resembled discharges seen in neuromyotonia, were detectable on EMG testing on days 2 to 4 post-treatment with the muscle clinically at rest independently of EMG needle insertion. They were detected in 100% of patients and 72% of muscles on days 2 to 4 after treatment with oxaliplatin plus capecitabine but in none of the patients or muscles tested before oxaliplatin treatment or after treatment with carboplatin plus paclitaxel or cisplatin. Abnormal, spontaneous high frequency motor unit activity was more frequent on days 2 to 4 than on days 14 to 20 post-treatment, and was more sensitive and more specific than repetitive CMAPs for detecting acute oxaliplatin-induced motor nerve hyperexcitability. Repetitive CMAPs were abnormal action potentials occurring after a single electrical stimulus following the main CMAP. These findings confirm a previous report [5,6] of spontaneous high frequency motor unit action potentials and repetitive CMAPs after oxaliplatin treatment and refined clinical procedures for the neurophysiological assessment of chemotherapy-induced motor nerve hyperexcitability.

The neurophysiological features of acute oxaliplatin-induced motor nerve hyperexcitability found in this study, and those described previously [5,6] are similar to those seen in acquired neuromyotonia. Neuromyotonia is often associated with autoantibodies against potassium channels [7], and it has been postulated that disruption of K^+ ^channels is a key element in the production of neuromyotonia. However, in a mouse diaphragm model, K^+ ^channel blockade did not replicate the neurophysiological features of oxaliplatin[8]. Instead, the increase in spontaneous activity induced by oxaliplatin in this model was prevented by the voltage-gated Na^+ ^channel blocker tetrodotoxin. The authors concluded that the most likely mechanism of oxaliplatin neurotoxicity is a direct effect on voltage-gated Na^+ ^channels rather than an effect on K^+ ^channels or nerve terminal Ca^2+ ^regulation. Others have also found evidence of Na^+ ^channel dysfunction with oxaliplatin use [9,10] although this has not been a consistent finding [11].

Repetitive CMAPs may be generated at the synapse or in the motor nerve axon [12]. Synaptic CMAPs may occur when there is excessive acetyl choline (ACh) in the synapse, and they can also occur with normal amounts of ACh in the synapse in the slow channel syndrome [13]. There is no reason to suppose that repetitive CMAPs in the setting of oxaliplatin use are due to excessive ACh in the synapse. Instead, the repetitive CMAPs that were observed following a single electrical stimulus were almost certainly generated in the motor nerve axons themselves. Repetitive CMAPs similar to those recorded here have been described in neuromyotonia,[12] and the authors concluded from their studies that the focus of abnormal excitation must have been in the distal part of the nerve. Presumably disruption of function of sodium channels is also the reason for the appearance of the repetitive CMAPs with oxaliplatin use, as well as causing the spontaneous high frequency motor unit activity seen on EMG.

Neurophysiological studies are more subjective than may be appreciated, and for this reason considerable lengths were taken to eliminate bias from the current study by including relevant control groups and ensuring the neurophysiologist was blinded to the treatment group and patient details. In particular, determining whether there is abnormal insertional EMG activity is very subjective, and this is likely to have contributed to false positive tests recorded in control patients. However, the more robust finding of abnormal spontaneous high frequency motor unit activity was not detected in any of the control patients who were tested before receiving oxaliplatin or after other chemotherapeutic agents. Similarly, it was difficult at times to determine whether there were one or more abnormal repetitive CMAPs following a single stimulus, and whether this had occurred because some patients were not adequately relaxed for testing. However, the impression was that repetitive CMAPs were much more prominent after oxaliplatin than in any other circumstance, but it was not possible to devise any method for quantifying this abnormality to improve its specificity.

Motor nerve hyperexcitability with subsequent abnormal muscle contraction could be a basis for some of the acute neurotoxicity symptoms reported by patients after oxaliplatin treatment. Approximately 70% of patients treated with oxaliplatin in the current study complained of jaw or throat tightness, and 4 of 22 patients reported muscle cramps. However, motor symptoms occurred less frequently than abnormal, spontaneous high frequency motor unit activity as determined by EMG assessment of subclinical motor nerve hyperexcitability. Cold-induced paraesthesiae were reported universally by oxaliplatin-treated patients in the current study, with or without other sensory symptoms, which may be due to sensory nerve hyperexcitability that was undetectable by conventional neurophysiological testing.

Park et al [9] found evidence of sensory nerve hyperexcitability and functional channelopathy of axonal sodium channels when they undertook axonal excitability studies in 58 patients who were treated with oxaliplatin. Significant changes were evident in both sensory and motor axons in recovery cycle parameters immediately following oxaliplatin infusions. The extent of oxaliplatin-induced abnormality in motor axons was proportional to the degree of change in sensory axons. Abnormalities became progressively worse in sensory axons, while motor axonal excitability remained unchanged with repeated doses of oxaliplatin. Sensory excitability abnormalities that developed during early treatment cycles were able to predict the development of chronic sensory neuropathy on an individual patient basis in 80% of patients, in their study. The neurophysiological studies undertaken by Park et al [9] were notably different from those performed in the current study. Their studies required specialised equipment, but our studies comprised conventional clinical neurophysiological studies, and included detailed EMG studies. As such, the studies are complementary. The testing in the current study, which was less sophisticated than that undertaken by Park et al [9], did not identify sensory abnormalities acutely, but found clear evidence of motor nerve excitability acutely that resolved over subsequent weeks.

Finally, our study suggests that the mechanism of acute oxaliplatin-induced motor nerve hyperexcitability differs from the mechanisms involved in the chronic neuro-sensory toxicities of oxaliplatin and related chemotherapeutic agents. We showed evidence of acute motor nerve hyperexcitability on nerve conduction studies and EMG after oxaliplatin treatment, but not following treatment with carboplatin plus paclitaxel or cisplatin. In contrast, cisplatin, carboplatin plus paclitaxel and oxaliplatin are well known for inducing chronic neuro-sensory toxicity in a high percentage of treated patients that typically present with chronic neuro-sensory symptoms and loss of sensation with motor function spared [2,3,14]. Differences between the acute and chronic neurotoxicity profiles of different chemotherapy drugs, and in their relative specificity for inducing toxicity to peripheral sensory versus motor nerves, suggests that these are distinct and mechanistically unrelated neurotoxicities. The mechanisms of acute and chronic neurotoxicities of platinum drugs are unknown. However, acute motor nerve hyperexcitability induced by oxaliplatin may be due to chelation of calcium or other endogenous divalent cations by its oxalate leaving group [15], but not by the leaving groups of other platinum drugs. In contrast, the chronic neuro-sensory toxicities of cisplatin, carboplatin and oxaliplatin maybe due to neuronal accumulation of platinum [16], which is a common element of all chemotherapeutic agents of the class.

## Conclusions

Abnormal, spontaneous high frequency motor fibre activity is a sensitive and specific endpoint of acute oxaliplatin-induced motor nerve hyperexcitability, detectable on EMG on days 2 to 4 post-treatment. Objective EMG assessment of motor nerve excitability could compliment patient-reported symptomatic endpoints of acute oxaliplatin-induced neurotoxicity in future studies, and is suitable for use in multicentre clinical trials as EMG is widely available as a routine diagnostic clinical procedure.

## Competing interests

The authors declare that they have no competing interests.

## Authors' contributions

AH coordinated patient recruitment and study procedures, and contributed to data interpretation and preparation of the final manuscript. PB contributed to the study conception, design, data interpretation and preparation of the final manuscript, and carried out all of the neurophysiological assessments. FH contributed to coordinating patient recruitment and study procedures. PT, MF and DD contributed patients. MM contributed to the study conception, design, coordination, patient recruitment, data interpretation and preparation of the final manuscript. All authors read and approved the final manuscript.

## Pre-publication history

The pre-publication history for this paper can be accessed here:

http://www.biomedcentral.com/1471-2407/10/451/prepub
